# First-in-human safety and pharmacokinetics of MK-7602, the antimalarial inhibitor of plasmepsins IX/X, in single- and multiple-ascending-dose studies

**DOI:** 10.1128/aac.01261-25

**Published:** 2026-01-14

**Authors:** Susan E. Stanley, Russ P. Carstens, Maria V. Liberti, Ward Eertmans, Myrthel Vranckx, Diane Longo, Nairita Ghosal, Marissa Vavrek, David B. Olsen, Alan F. Cowman, Tom Reynders, Caroline Cilissen, Tine Laethem, Sylvie Rottey, Jonathan A. Robbins, S. Aubrey Stoch, Jesse Nussbaum

**Affiliations:** 1 MRL, Merck & Co. 2793 , Rahway, New Jersey, USA; 2 MSD, Brussels, Belgium; 3 The Walter and Eliza Hall Institute of Medical Research, Parkville, Australia; 4 Ghent University, Ghent University Hospital 26656, Ghent, Belgium; The Children's Hospital of Philadelphia, Philadelphia, Pennsylvania, USA

**Keywords:** antimalarial drug, plasmepsin IX, plasmepsin X, malaria, MK-7602

## Abstract

MK-7602 is a first-in-class dual-plasmepsin inhibitor being developed to treat malaria. Safety, tolerability, and pharmacokinetics (PK) of MK-7602 following single and multiple doses were evaluated in two phase 1 studies (7602-001; 7602-002). Study 7602-001 included two parts: part 1, a randomized, single-ascending-dose (10–400 mg), placebo-controlled, double-blind study (*n* = 24); and part 2, a non-randomized, fixed-sequence, open-label study (*n* = 12) to assess the effect of itraconazole (200 mg), a cytochrome P450 3A and P-glycoprotein inhibitor, on the PK of MK-7602 (25 mg). Study 7602-002 was a randomized, placebo-controlled, multiple-ascending-dose study (*n* = 40); participants received MK-7602 (50–300 mg) or placebo for 7 days. Single and multiple doses of MK-7602 were generally well tolerated. Headaches were the most common adverse event (7602-001 part 1: 54.5%; 7602-002: 36.7%). MK-7602 median time to maximal concentration (*T*_max_) was 1.5–3.0 h, with dose-proportional increases in maximum concentration (*C*_max_) and the area under the curve over the dosing interval (AUC_0-tau_) at single and multiple doses of ≥50 mg. Terminal half-life was 31.3–41.4 h following multiple dosing, the accumulation ratio for daily dosing was 1.03–2.20, and steady-state concentrations were reached by day 3. Coadministration with itraconazole resulted in a 6- and 12-fold increase in *C*_max_ and area under the concentration-time curve to infinity, respectively. The primary hypothesis that a well-tolerated dose of MK-7602 would achieve a trough concentration of ≥0.017 μM was met in both studies. Safety and PK characteristics support continued development of MK-7602.

## INTRODUCTION

*Plasmodium falciparum*, the parasite responsible for the most severe form of malaria in humans, accounts for over 90% of the global malaria burden, with the highest prevalence in Africa (1–3). *P. vivax* and *P. knowlesi* infections can also lead to severe disease, although less frequently (4). *P. falciparum* has developed resistance to all major classes of antimalarial drugs, including reduced susceptibility to first-line artemisinin-based combination therapies in certain regions of the world (5, 6). This increasing resistance poses a significant threat to global malaria control and highlights the urgent need for antimalarial drugs with novel mechanisms of action (5).

**Fig 1:**
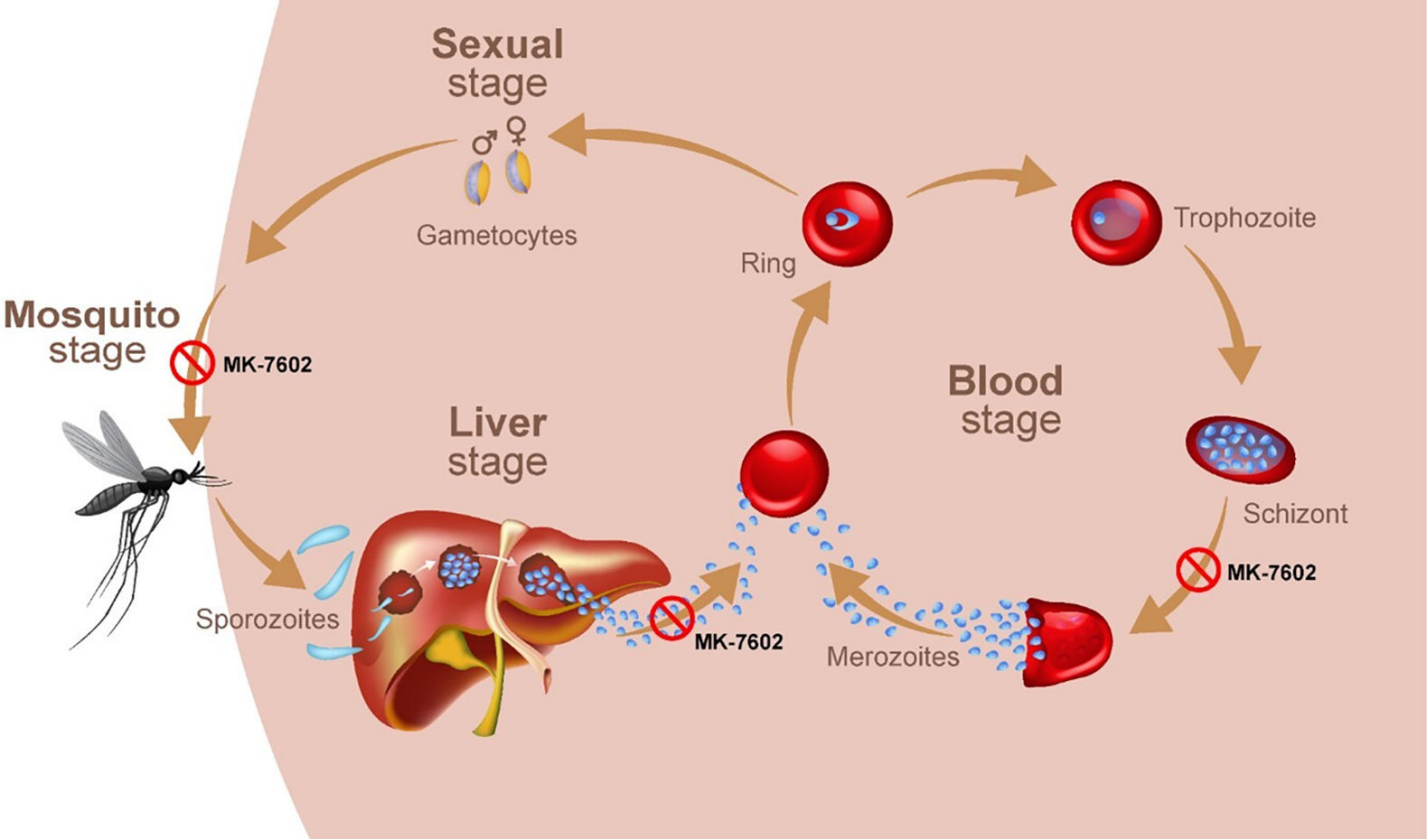
An overview of the malaria parasite life cycle demonstrating the three-stage inhibition potential of MK-7602. The biochemical targets for MK-7602, plasmepsin IX (PMIX), and plasmepsin X (PMX) are expressed in the liver, blood, and sexual (transmission or mosquito) stages of the replication cycle.

MK-7602 is a novel, dual-plasmepsin inhibitor being developed for the treatment of acute uncomplicated *P. falciparum* malaria. MK-7602 targets the conserved aspartic proteases plasmepsin IX (PMIX) and plasmepsin X (PMX) in *Plasmodium* spp., disrupting parasite replication across multiple stages of its life cycle (7–11) (Fig. 1). PMIX and PMX are aspartic proteases essential to the maturation of functional merozoites in the liver. They facilitate merozoite egress and invasion of erythrocytes in the blood, and their activity is critical for gametocyte development and transmission to mosquitoes during the sexual stage (8)*.* Given their functional importance in *Plasmodium* spp. and the lack of homologous proteases in humans, PMIX and PMX are appealing targets for antimalarial drug development (8).

In preclinical studies, MK-7602 exhibited potent activity against *P. falciparum* (half-maximal effective concentration [EC_50_] of 0.40 nM in a lactate dehydrogenase–based assay), *P. knowlesi, P. berghei*, and *P. vivax*, demonstrating its potential as a broad-spectrum antimalarial agent (11). Importantly, MK-7602 showed no cross-resistance with *P. falciparum* strains that are resistant to other antimalarials, including chloroquine, mefloquine, atovaquone, and artemisinin (10, 11).

This article describes the initial findings from two phase 1 studies (7602-001 and 7602-002) conducted to evaluate the safety, tolerability, and pharmacokinetics (PK) of MK-7602 in healthy adults.

## MATERIALS AND METHODS

### Study design

Studies 7602-001 (EudraCT: 2022-001845-19) and 7602-002 (EU CT: 2023-503738-36-00) were single-center (Drug Research, Ghent, Belgium) phase 1 studies conducted to evaluate the safety, tolerability, and PK of single- and multiple-ascending doses of MK-7602 in healthy adults.

#### Overview of study 7602-001

**Fig 2:**
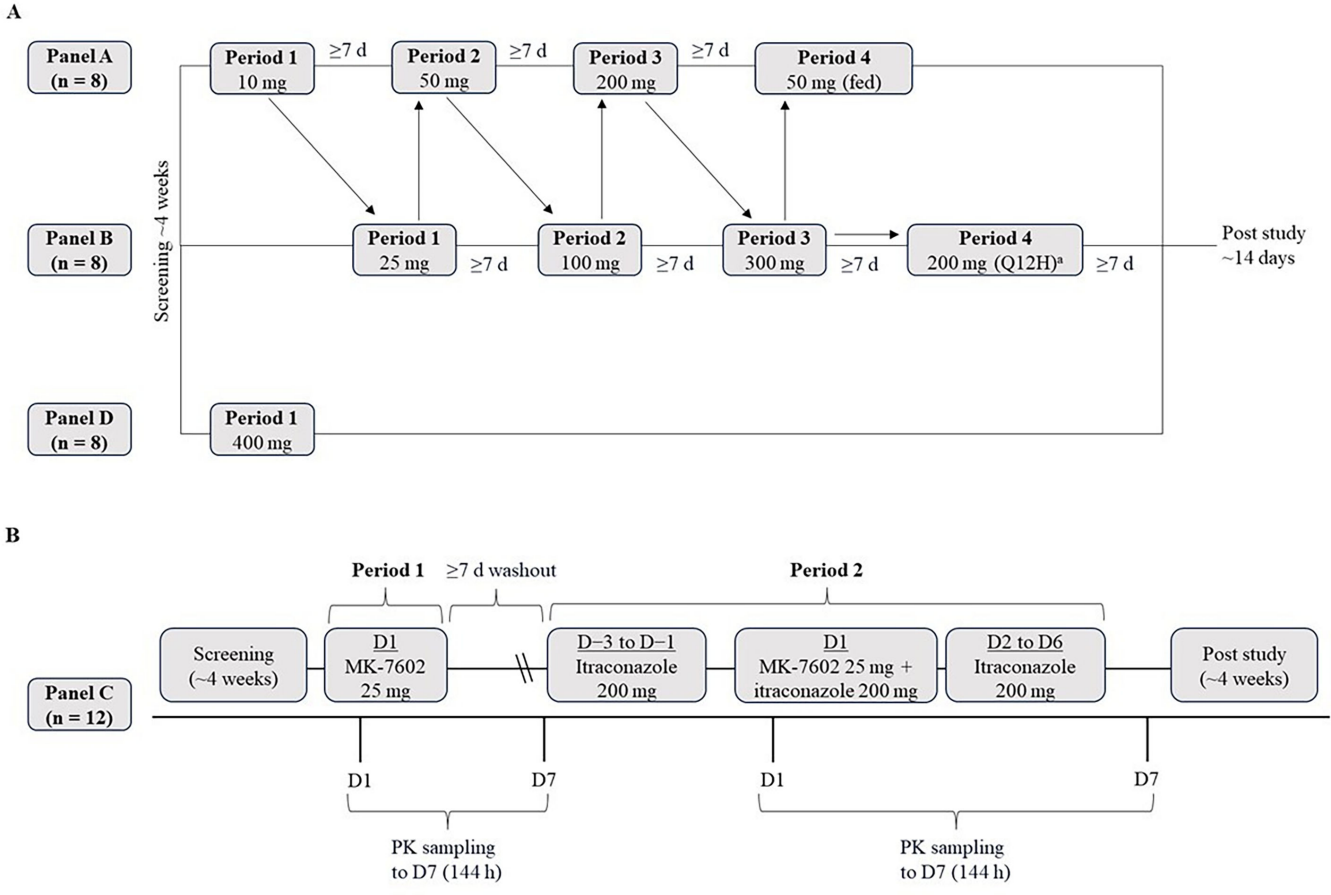
Study design of 7602-001. (**A**) Part 1 and (**B**) part 2. All doses of MK-7602 were administered as single doses, except for in panel B, period 4. Study 7602-001 part 1 included three panels—A, B, and D—in all of which participants were randomized (6:2) to receive MK-7602 (*n* = 6) or placebo (*n* = 2). Study 7602-001 part 2 was conducted with panel C as an open-label panel (*n* = 12). ^a^MK-7602 400 mg was administered as a split dose of two doses of 200 mg Q12H. PK, pharmacokinetics; Q12H, every 12 h; Q24H, every 24 h.

Study 7602-001 was divided into two parts (part 1 and part 2; Fig. 2). Part 1 was a randomized, placebo-controlled, double-blind, alternating-panel study with single-ascending doses in panels A, B, and D (Fig. 2A). Part 2 of 7602-001 was a non-randomized, fixed-sequence, open-label study consisting of panel C (Fig. 2B).

Part 1 consisted of four treatment periods for panels A and B, while panel D consisted of a single period. In panels A and B (*n* = 8 in each), participants were randomized to receive ascending oral doses of MK-7602 (*n* = 6) ranging from 10 to 400 mg or placebo (*n* = 2) in alternating periods. All doses in part 1 were administered as single doses, except for the 400 mg dose in panel B, period 4, which was given as a split dose (200 mg every 12 h [Q12H]) to ensure that the concentrations remained below the protocol-defined maximum limit. Panel A received MK-7602 50 mg or placebo in the fasted state in period 2 and MK-7602 50 mg or placebo after a high-fat meal in period 4 (dietary details are provided in the supplemental material). Panel D, comprised of eight participants, was added to evaluate a single dose of MK-7602 400 mg (*n* = 6) compared with placebo (*n* = 2).

In part 2, period 1, participants (*n* = 12) received a single dose of MK-7602 25 mg as the PK reference. In period 2, the same participants received itraconazole 200 mg every 24 h (Q24H) as an oral solution from day −3 to day −1. On day 1, itraconazole 200 mg was administered approximately 1 h prior to a dose of MK-7602 25 mg, which was given in the morning after an 8-hour fast. Participants remained fasting for 4 h following administration of MK-7602. From days 2 to 6, itraconazole 200 mg alone was administered Q24H. Itraconazole was administered with or without food, except when fasting was required for PK evaluations.

#### Overview of study 7602-002

**Fig 3:**
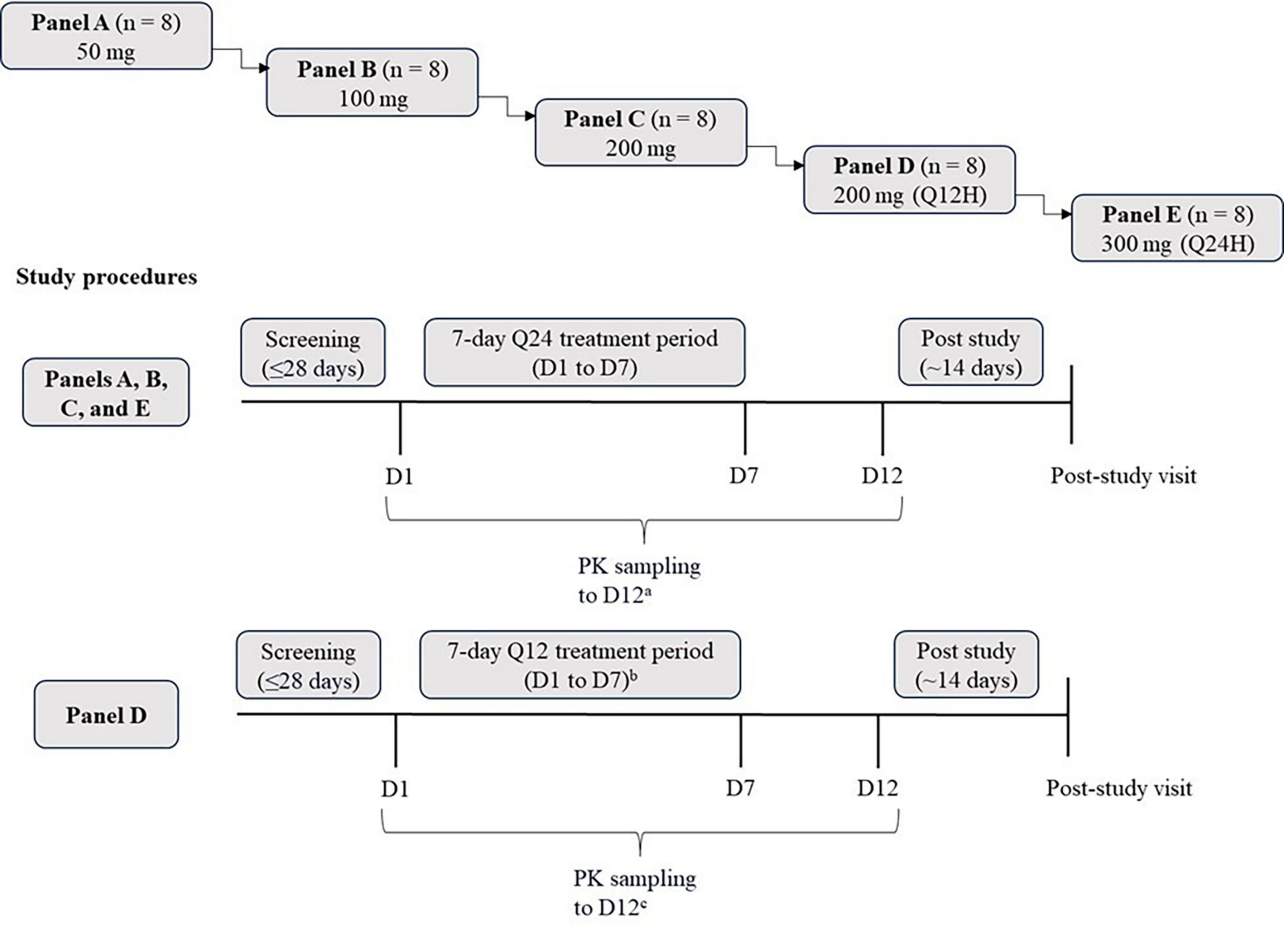
Study design of 7602-002. Each panel had eight participants randomized (3:1) to receive MK-7602 (*n* = 6) or placebo (*n* = 2). ^a^Intensive PK sampling occurred on day 1, day 3 (panel A only), and day 7. ^b^Day 7 intervention was administered in the morning. ^c^Intensive PK sampling occurred on day 1, day 3, and day 7 for panel D. PK, pharmacokinetics; Q12H, every 12 h; Q24H, every 24 h.

Study 7602-002 was a randomized, placebo-controlled, multiple-ascending-dose, double-blind study consisting of panels A, B, C, D, and E (Fig. 3). In panels A, B, C, and E (*n* = 8 in each), participants were randomized to receive oral doses of MK-7602 (*n* = 6) ranging from 50 to 300 mg or placebo (*n* = 2) Q24H for 7 consecutive days. An additional panel (panel D) of eight participants received oral doses of MK-7602 200 mg (*n* = 6) or placebo (*n* = 2) twice daily (Q12H) for 6.5 consecutive days. The final dose was administered in the morning on day 7. Each participant was eligible to participate in only one panel.

### Study procedures and safety monitoring

Safety was monitored throughout each study through evaluation of vital signs, 12-lead electrocardiography (ECG), laboratory values, physical examination, and adverse events (AEs). In 7602-001, parts 1 and 2, participants received MK-7602 or placebo after an 8-hour fast, except when administered after a high-fat meal in panel A, period 4. Serial blood samples for MK-7602 plasma PK were collected pre-dose and at 0.25, 0.5, 1, 1.5, 2, 3, 4, 6, 8, 12, 16, 24, 48, 72, 96, and 120 h post dose in parts 1 and 2, including at 144 h following each MK-7602 dose in part 2. In part 1 of 7602-001, urine PK samples were collected from panel A, period 1 (50 mg in the fasted state) and panel B, period 3 (300 mg).

In 7602-002, participants in panels A, B, C, and E received MK-7602 or placebo daily after an 8-hour fast, whereas in panel D (Q12H administration), participants fasted for 8 h prior to the morning dose and for 2 h prior to the evening dose. Serial blood samples for MK-7602 plasma PK were collected pre-dose and at 0.5, 1, 1.5, 2, 3, 4, 6, 8, and 12 h post dose on days 1 and 7, with sampling at 24, 48, 72, 96, and 120 h post dose following the final dose on day 7. In panels A and D, intensive PK sampling additionally took place on day 3. Safety evaluations—including vital signs, physical examinations, laboratory evaluations, and ECG—were conducted throughout the dosing interval, concentrated around predicted *T*_max_ (~2 h post dose) and taking into consideration the effective terminal half-life (~12 h) and an apparent terminal half-life (~23 h). All dose-escalation decisions were made jointly by the investigator and the sponsor, based on review of blinded safety and tolerability data and available PK data from the previous dosing period.

### Study participants

Study 7602-001 included healthy adults aged 18–45 years with a body mass index (BMI) of 18–30 kg/m². Only adult male participants were enrolled, as this was the initial administration of MK-7602 to humans, and no *in vivo* developmental or reproductive toxicity studies were performed prior to study start. Study 7602-002 included healthy participants of non-childbearing potential aged 18–55 years with a BMI of 18–32 kg/m². Female participants were considered to be of non-childbearing potential if they had undergone documented hysterectomy, bilateral salpingectomy, or bilateral oophorectomy, or if they were postmenopausal. Male participants were asked to refrain from donating sperm, abstain from sex, or use contraception for 90 days after study intervention, unless they were confirmed to be azoospermic. Participants were in good health, based on their medical history, physical examination, laboratory values, vital sign measurements, and ECG. Key exclusion criteria included a history of clinically significant medical conditions and, for part 2 of 7602-001, a history of hypersensitivity to itraconazole or other azole antifungal agents. The full list of exclusion criteria is shown in Table S1.

### Bioanalysis methods

Plasma and urine MK-7602 concentrations were determined by a validated liquid chromatography tandem-mass spectrometry assay at Pharma Medica Research Inc., Ontario, Canada. Laboratory safety assessments (hematology, chemistry, and urinalysis) were conducted at the Ghent University Hospital, Ghent, Belgium.

### Primary hypothesis

The primary hypothesis in these studies was that the true geometric mean trough concentration (*C*_trough_) of MK-7602 was ≥0.017 μM on day 1, following a well-tolerated single dose of MK-7602 in study 7602-001, and on day 1 and day 7, following multiple doses in study 7602-002. The plasma *C*_trough_ of 0.017 μM corresponds to 5× the unbound *in vitro* EC_50_ of 0.40 nM, a target that was associated with efficacy in *P. berghei* mouse models (11). For the Q12H dosing regimen, the hypothesis applied to *C*_trough_ measured at the end of the 12-hour interdose interval (just before the second dose), rather than 24 h post dose. The selection of doses in these studies was based on the likely therapeutic range based on preclinical data.

### Statistical and analysis methods

#### Model-based PK summary

In 7602-001, for each PK parameter (area under the concentration-time curve to infinity [AUC_0-∞_], maximum concentration [*C*_max_], and concentration at 24 h [*C*_24_]), individual values at each fasted and fed dose level were natural log-transformed. These were evaluated with a linear mixed-effects model containing a fixed effect for treatment and a random effect for participant. The Kenward-Roger method was used to calculate the denominator degrees of freedom (den*df*) for the fixed effects. In 7602-002, plasma MK-7602 AUC over the dosing interval (AUC_0-tau_), *C*_max_, and *C*_trough_ were natural log-transformed and evaluated using separate linear mixed-effects models containing fixed effects for treatment, day (day 1, day 7), treatment-by-day interaction, and a random effect for participant.

For both studies, least squares (LS) means for each treatment were derived on the logarithmic scale to estimate population geometric means (GMs) and to construct 95% confidence intervals (CIs), referenced to the t-distribution. The LS means and the lower and upper limits of the CIs were exponentiated to obtain estimates for the population GMs and 95% CIs on the original scale. In 7602-002, LS mean differences (days 7–1) and 90% CIs from this model were back-transformed to obtain the GM accumulation ratio (day 7/day 1) and 90% CIs for plasma MK-7602 AUC_0-tau_ and *C*_max_.

#### Target PK concentration

The primary hypothesis was tested using Bayesian methodology based on the LS estimates from the linear mixed-effects model. The posterior probability that the true GM was ≥0.017 μM was calculated for each dose, assuming normality and a non-informative prior. A prespecified threshold of 70% for the posterior probability at least one dose level that also exhibited an acceptable safety and tolerability profile satisfied the PK hypothesis.

#### Dose proportionality

An exploratory assessment of steady-state dose proportionality of plasma MK-7602, AUC_0-tau_, *C*_max_, and *C*_trough_ was conducted in study 7602-002 using the power law model for Q24H dosing panels. Separately for each PK parameter, individual PK values on day 7 were natural log-transformed and evaluated using a linear regression model with ln(dose) as the explanatory variable. An LS mean estimate and 95% CI for the slope associated with ln(dose) were obtained from the model.

#### Drug-drug interactions

In 7602-001, for each PK parameter (AUC_0-∞_, *C*_max_, and *C*_24_) of MK-7602 25 mg, individual values were natural log-transformed and analyzed using a linear mixed-effects model, with treatment (MK-7602 alone or with itraconazole 200 mg Q24H) as a fixed effect and participant as a random effect. The Kenward-Roger method was used for calculating the den*df*. A 90% CI for the difference in LS means was constructed on the log scale and exponentiated to determine the true GM ratio (GMR). A 95% CI was also constructed for the GMs for each treatment based on the PK model. Additionally, plots depicting the individual ratios, GMR, and the 90% CI were provided for AUC*_0_*_-∞_ and *C*_max_.

#### Food effect

The effect of food on MK-7602 was assessed in 7602-001 by calculating the GM ratio (GMR) and 90% CI for AUC_0-∞_, *C*_max_, and *C*_24_, using the linear mixed-effects model for 50 mg doses administered in both the fed and the fasted states. AUC_0-24_ was also analyzed.

## RESULTS

### Participant disposition

In 7602-001 part 1, 24 participants were randomized across three panels (eight participants per panel, randomized 6:2 to receive MK-7602 or placebo). The placebo allocation was rotated among the eight participants in each period, resulting in 22 participants receiving MK-7602 and 18 receiving placebo. In part 2, 12 participants were assigned to treatment. All randomized participants (*n* = 36) completed the study. In total, 36 participants were included in the population for safety analysis, and 34 participants were included in the population for PK analysis (22 for part 1 and 12 for part 2). One participant in panel A missed a dose (200 mg) in period 3 due to coronavirus 2019, which began on day −6 of period 3.

In 7602-002, 40 participants were randomized and treated (eight in each panel, randomized 6:2 such that 30 received MK-7602 and 10 received placebo). A total of 39 participants completed their assigned treatment, and 40 participants completed the study. All 40 participants were included in the safety analyses, and the 30 who received MK-7602 were included in the PK analysis. One participant in panel E discontinued the study intervention on day 5 after receiving four doses of MK-7602 300 mg Q24H due to a moderate AE of burning sensation in the face and neck, which was accompanied by mild erythema and pruritus (see Safety analysis section for further details).

### Demographics and baseline characteristics

Overall, 94.4% of participants in 7602-001 and 92.5% in 7602-002 were White. In 7602-002, 77.5% were male. The mean age of participants was 30.8 years (standard deviation [SD]: 8.3; range: 19–44 years) in 7602-001 and 33.3 years (SD: 11.8; range: 18-45) and 43.9 years (SD: 13.8; range: 18–45) in the MK-7602 and placebo groups, respectively, in 7602-002. The mean BMI was 23.7 kg/m² (SD: 2.8; range: 17.6–30.1 kg/m²) in 7602-001, and 23.9 kg/m² (SD: 3.0; range: 19.9–30.0) and 25.3 kg/m² (SD: 3.7; range: 19.1–29.2) in the MK-7602 group and placebo group, respectively, in 7602-002 (Tables 1 and 2).

**TABLE 1:** Participant demographics and baseline characteristics in 7602-001^*a*^^,^^*b*^

	Panel A *n* = 8	Panel B *n* = 8	Panel C *n* = 12	Panel D *n* = 8	Total *N* = 36
Age, years					
Mean ± SD	29.9 ± 7.9	30.5 ± 7.4	33.0 ± 9.4	28.5 ± 8.5	30.8 ± 8.3
Median	30	33	32.5	27	30
Range	19–43	19–41	22–44	19–42	19–44
Race, *n* (%)					
Asian	0	0	0	1 (12.5)	1 (2.8)
Black or African American	0	0	1 (8.3)	0	1 (2.8)
White	8 (100)	8 (100)	11 (91.7)	7 (87.5)	34 (94.4)
Ethnicity, *n* (%)					
Not Hispanic or Latino	8 (100)	8 (100)	12 (100)	8 (100)	36 (100)
Height, cm					
Mean ± SD	177.7 ± 4.8	181.2 ± 4.4	177.7 ± 5.9	179.1 ± 6.1	178.8 ± 5.4
Median	177.3	181	177.2	179.1	178.2
Range	168.7–183.5	174.3–186.7	165.5–186.4	171.0–186.5	165.5–186.7
Weight, kg					
Mean ± SD	74.5 ± 8.4	80.2 ± 9.0	75.6 ± 12.0	72.9 ± 12.8	75.8 ± 10.7
Median	73.8	78.5	76.2	69.6	73.8
Range	62.8–90.4	69.8–94.4	48.2–90.6	60.8–95.8	48.2–95.8
BMI, kg/m^2^					
Mean ± SD	23.6 ± 1.8	24.5 ± 2.6	23.8 ± 2.7	22.8 ± 4.7	23.7 ± 2.8
Median	23.4	24.2	23.9	21.5	23.4
Range	20.7–26.8	20.7–28.1	17.6–27.1	18.9–30.1	17.6–30.1

^*a*^ BMI, body mass index; Q12H, every 12 h; SD, standard deviation.

^*b*^ All participants were male. Doses for each panel were as follows: panel A: placebo and MK-7602 10 mg, 50 mg, 200 mg, and 50 mg (with food); panel B: placebo and MK-7602 25 mg, 100 mg, 300 mg, and a 400 mg split dose (200 mg Q12H); panel C: MK-7602 25 mg and itraconazole 200 mg; panel D: placebo and MK-7602 400 mg.

**TABLE 2:** Participant demographics and baseline characteristics for 7602-002^*a*^

	MK-7602 50 mg Q24H *n* = 6	MK-7602 100 mg Q24H *n* = 6	MK-7602 200 mg Q24H *n* = 6	MK-7602 200 mg Q12H *n* = 6	MK-7602 300 mg Q24H *n* = 6	MK-7602 total *n* = 30	Placebo *n* = 10	Total *N* = 40
Sex, *n* (%)								
Male	5 (83.3)	6 (100)	4 (66.7)	5 (83.3)	5 (83.3)	25 (83.3)	6 (60.0)	31 (77.5)
Female	1 (16.7)	0 (0.0)	2 (33.3)	1 (16.7)	1 (16.7)	5 (16.7)	4 (40.0)	9 (22.5)
Age, years								
Mean ± SD	33.5 ± 14.1	34.7 ± 10.0	33.7 ± 16.5	32.7 ± 10.9	32.0 ± 10.4	33.3 ± 11.8	43.9 ± 13.8	36.0 ± 13.0
Median	26.0	36.5	27.0	27.5	29.0	28.0	50.0	30.0
Range	21–53	21–48	19–55	24–47	22–52	19–55	20–55	19–55
Race, *n* (%)								
Asian	0	0	0	0	0	0	1 (10.0)	1 (2.5)
Black or African American	0	0	1 (16.7)	0	1 (16.7)	2 (6.7)	0	2 (5.0)
White	6 (100)	6 (100)	5 (83.3)	6 (100)	5 (83.3)	28 (93.3)	9 (90.0)	37 (92.5)
Ethnicity, *n* (%)								
Not Hispanic or Latino	6 (100)	6 (100)	6 (100)	6 (100)	6 (100)	30 (100)	10 (100)	40 (100)
Height, cm								
Mean ± SD	182.1 ± 8.4	178.4 ± 6.7	169.9 ± 8.2	177.6 ± 13.6	180.4 ± 6.5	177.7 ± 9.4	171.1 ± 6.5	176.0 ± 9.2
Median	185.4	176.0	170.3	179.0	182.5	177.4	172.9	176.0
Range	169.2–190.0	172.1–188.2	159.7–178.5	158.0–192.0	169.4–186.3	158.0–192.0	161.0–179.5	158.0–192.0
Weight, kg								
Mean ± SD	80.1 ± 14.5	74.1 ± 11.5	69.4 ± 10.5	73.3 ± 11.0	82.6 ± 16.4	75.9 ± 13.0	74.0 ± 11.5	75.4 ± 12.5
Median	77.7	70.6	68.3	72.4	87.7	72.5	74.6	73.2
Range	64.4–103.6	65.4–96.6	54.2–81.2	60.2–90.8	62.6–98.8	54.2–103.6	58.4–90.8	54.2–103.6
BMI, kg/m^2^								
Mean ± SD	24.1 ± 3.6	23.2 ± 2.1	24.0 ± 3.5	23.3 ± 2.5	25.2 ± 3.6	23.9 ± 3.0	25.3 ± 3.7	24.3 ± 3.2
Median	23.5	22.3	23.3	23.3	25.9	23.1	26.6	24.0
Range	20.3–29.6	21.7–27.3	20.4–30.0	19.9–25.9	20.2–28.6	19.9–30.0	19.1–29.2	19.1–30.0

^*a*^ MK-7602 total column is pooled across panels A, B, C, D, and E. Placebo column is pooled across panels A, B, C, D, and E. BMI, body mass index; Q12H, every 12 h; Q24H, every 24 h; SD, standard deviation.

### Safety analysis

MK-7602 was well tolerated following single- and multiple-dose administration. In 7602-001, AEs were reported in a total of 91.7% of participants (22/24 in part 1 and 11/12 in part 2); in 90.9% (20/22) following administration of MK-7602; in 68.8% (11/16) following placebo in part 1; in 58.3% (7/12) following administration of MK-7602 alone (25 mg); and in 83.3% (10/12) during itraconazole + MK-7602 cotreatment (200 mg Q24H + 25 mg, respectively) in part 2. In 7602-002, AEs were reported in a total of 77.5% participants (31/40); in 80.0% (24/30) following administration of MK-7602; and in 70.0% (7/10) following placebo.

AEs experienced by more than one participant receiving MK-7602 included headache (54.5% [*n* = 12] in 7602-001 part 1 and 36.7% [*n* = 11] in 7602-002), oropharyngeal pain (27.3% [*n* = 6] in 7602-001 part 1 and 16.7% [*n* = 5] in 7602-002), and contact dermatitis (27.3% [*n* = 6] in 7602-001 part 1 and 30.0% [*n* = 9] in 7602-002). Diarrhea occurred in four participants (18.2%) in 7602-001 part 1, seven participants (58.3%) in 7602-001 part 2, and three participants (10.0%) in 7602-002. Nasopharyngitis was experienced by four participants (18.2%) in 7602-001 part 1 and by three participants (10.0%) in 7602-002. Abdominal pain was experienced by two participants (16.7%) in 7602-001 part 2. Other AEs included neck pain (18.2% [*n*=4] in 7602-001 part 1) and postural orthostatic tachycardia syndrome (9.1% [*n*=2] in 7602-001 part 1). The summary of AEs is shown in Tables S2 and S3 for 7602-001 and in Table 3 for 7602-002.

**TABLE 3:** Summary of AEs in 7602-002^*a*^^,^^*d*^

	MK-7602 50 mg Q24H	MK-7602 100 mg Q24H	MK-7602 200 mg Q24H	MK-7602 200 mg Q12H
Participants in population, *n*	6	6	6	6
With one or more AEs	5 (83.3)	5 (83.3)	3 (50.0)	5 (83.3)
With no AE	1 (16.7)	1 (16.7)	3 (50.0)	1 (16.7)
With drug-related AEs^*a*^	1 (16.7)	2 (33.3)	0	1 (16.7)
With non-serious AEs	5 (83.3)	5 (83.3)	3 (50.0)	5 (83.3)
With dose modification due to an AE^*b*^	0	0	0	0
Who discontinued drug due to an AE	0	0	0	0
Who discontinued drug due to a drug-related AE	0	0	0	0

	MK-7602 300 mg Q24H	MK-7602 total	Placebo	Total
Participants in population, *n*	6	30	10	40
With one or more AEs	6 (100)	24 (80.0)	7 (70.0)	31 (77.5)
With no AE	0	6 (20.0)	3 (30.0)	9 (22.5)
With drug-related AEs^*b*^	1 (16.7)	5 (16.7)	0	5 (12.5)
With non-serious AEs	6 (100)	24 (80.0)	7 (70.0)	31 (77.5)
With dose modification due to an AE^*c*^	1 (16.7)	1 (3.3)	0	1 (2.5)
Who discontinued drug due to an AE	1 (16.7)	1 (3.3)	0	1 (2.5)
Who discontinued drug due to a drug-related AE	1 (16.7)	1 (3.3)	0	1 (2.5)

^*a*^ AE, adverse event; Q12H, every 12 h; Q24H, every 24 h.

^*b*^ Determined by the investigator to be related to the drug.

^*c*^ Defined as an action taken of dose reduced, drug interrupted, or drug withdrawn.

^*d*^ Values are expressed as *n* (%) unless otherwise noted. No participants experienced serious AEs, serious drug-related AEs, died during the study, died due to a drug-related AE, discontinued the drug due to a serious AE, or discontinued the drug due to a serious drug-related AE.

Overall, AEs considered drug-related by the investigator were reported in 45.8% (11/24) and 66.7% (8/12) of participants in 7602-001 part 1 and part 2, respectively; 45.5% (10/22) following administration of MK-7602 and 18.8% (3/16) following placebo in part 1; 41.7% (5/12) following administration of MK-7602 alone; and 50.0% (6/12) following coadministration with itraconazole in part 2. One participant experienced a severe AE of diarrhea in part 2, period 2, during the itraconazole dosing period. Mild diarrhea started on day −3, became severe by day −2, and continued as mild through day 14. The diarrhea was assessed as not related to MK-7602 or itraconazole. Seventeen events of diarrhea were reported during coadministration, and none were reported with MK-7602 alone. In 7602-002, drug-related AEs were reported in 12.5% of participants (5/40); in 16.7% (5/30) following administration of MK-7602; and in 0 (0/10) following placebo.

There were no discontinuations due to AEs in 7602-001. One participant in 7602-002 discontinued due to a moderate AE of burning sensation in the face and neck, accompanied by mild erythema and pruritus. The symptoms started on day 5, after receiving four doses of MK-7602 300 mg Q24H. The erythema was mild and resolved after 1 week, and the burning sensation became mild after 5 days. By the end of the study, both the burning sensation and pruritus were mild and stabilized. All AEs experienced by this participant were assessed by the investigator to be related to the study intervention. Laboratory assessments of erythrocyte sedimentation rate and C-reactive protein at the time of symptoms were within normal limits. Participant medical history was notable for oral allergy syndrome, hay fever, contact allergy to cobalt and nickel, and seasonal allergic conjunctivitis. A subsequent dermatology consult diagnosed the participant’s symptoms as chronic urticaria. No serious AEs or deaths were reported in these studies in either intervention group, and no clinically significant trends were observed in vital signs, ECGs, laboratory safety values, or AEs or AE severity as a function of MK-7602 intervention or dose.

### Concentration/primary hypothesis

In 7602-001, *C*_trough_ of ≥0.017 μM was achieved at doses of MK-7602 between 50 and 400 mg (Table S4). After the 400 mg split dose, *C*_24_ values exceeded those of any single doses (Table 4). In 7602-002, the probability that GM *C*_trough_ on days 1 and 7 exceeded 0.017 μM was >70% for doses of 100, 200, and 300 mg Q24H (Table S5), as well as 200 mg Q12H (Table S6).

**TABLE 4:** Summary geometric mean (GCV) PK parameter values for MK-7602 in plasma following administration of oral single-ascending doses of MK-7602 in 7602-001^*a*^

Panel	Period	Dose, mg	*n*	AUC_0-∞_, µM·h^*b*^	AUC_0-last_, µM·h	AUC_0-24_, µM·h	*C*_max,_ µM	*C*_24,_ µM	*T*_max_, hours^*c*^	*t*₁/₂, hours	CL/F, L/h
A	1	10	6	NR	0.0340 (134.6)^*d*^^,^^*e*^	0.0277 (158.7)	0.00925 (96.5)	NR	1.81 (1.03–4.08)	NR	NR
B	1	25	6	0.280 (60.5)	0.229 (50.1)	0.213 (43.5)	0.0543 (64.9)	0.00315 (55.8)^*f*^	2.54 (1.03–4.08)	12.8 (99.3)	171 (60.5)
C	1	25	12	0.237 (90.0)	0.152 (84.0)	0.147 (68.7)	0.0347 (103.3)	0.00228 (68.0)^*e*^	2.08 (0.50–6.00)	17.7 (144.7)	203 (90.0)
C	2	25^*g*^	12	2.95 (34.3)	2.44 (37.2)	1.27 (44.6)	0.222 (51.5)	0.0286 (31.2)	1.50 (1.00–2.13)	75.1 (30.3)	16.3 (34.3)
A	2	50	6	0.706 (24.7)	0.624 (27.5)	0.481 (23.4)	0.115 (12.4)	0.00715 (23.3)	2.54 (0.55–4.08)	23.2 (9.4)	136 (24.7)
A	4	50 (fed)^*h*^	6	0.289 (98.8)	0.214 (101.5)	0.192 (77.3)	0.0325 (124.6)	0.00442 (65.1)^f^	3.57 (1.03–6.00)	14.7 (92.6)	333 (98.8)
B	2	100	6	2.46 (55.7)	2.30 (60.4)	1.71 (70.4)	0.466 (68.8)	0.0228 (34.1)	2.54 (1.07–4.08)	38.9 (27.6)	78.2 (55.7)
A	3	200	5	6.90 (37.1)	6.66 (37.2)	5.25 (39.6)	1.24 (20.9)	0.0545 (41.6)	2.08 (1.03–3.00)	41.2 (14.0)	55.7 (37.1)
B	3	300	6	5.69 (76.1)	5.50 (79.2)	4.22 (101.9)	0.971 (80.9)	0.0437 (48.4)	2.08 (1.03–3.00)	36.0 (17.0)	101 (76.1)
B	4	200 (Q12H)^*i*^	6	8.86 (32.0)	8.61 (32.1)	5.49 (40.2)	0.662 (29.9)	0.184 (32.5)	8.52 (1.03–16.00)	29.4 (22.8)	86.7 (32.0)
D	1	400	6	8.98 (101.5)	8.83 (103.8)	7.50 (116.6)	1.62 (89.2)	0.0551 (76.0)	2.08 (1.50–4.08)	30.6 (16.6)	85.5 (101.5)

^*a*^ %CV, percentage coefficient of variation; %GCV, percentage geometric coefficient of variation; AUC_0-24_, area under the curve from time 0 to 24 h; AUC_0-∞_, area under the concentration-time curve to infinity; AUC^_0-last_^, area under the curve from time zero to the last measurable concentration; BLOQ, below the limit of quantification; *C*_24_, concentration at 24 h; CI, confidence interval; CL/F, apparent clearance; *C*_max_, maximum concentration; LLOQ, lower limit of quantification; NR, not reported; PK, pharmacokinetics; Q12H, every 12 h; QD, once daily; *t*₁/₂, half-life; *T*_max_, time to maximum concentration.

^*b*^ The number of participants with AUC% extrapolated values ≥25% was as follows: *n* = 3 in panel A of period 1, *n* = 1 in panel B of period 1, *n* = 7 in panel C of period 1, and *n* = 2 each in panel C of period 2 and panel A of period 4.

^*c*^ Median (minimum, maximum) reported for *T*_max_ (hours).

^*d*^ *n *= 5.

^*e*^ One participant had only two measurable concentrations available, and the others were BLOQ. Therefore, AUC_0-last_ is not reported.

^*f*^ The number of participants with no measurable concentrations (BLOQ) at *C*_24_ was as follows: one of six in panel B of period 1, one of six in panel A of period 4, and three of 12 in panel C of period 1.

^*g*^ In period 2 of panel C, participants received MK-7602 25 mg in combination with itraconazole 200 mg QD.

^*h*^ In period 4 of panel A, participants received MK-7602 50 mg after a standard high-fat breakfast.

^*i*^ In period 4 of panel B, participants received MK-7602 400 mg in divided doses (200 mg Q12H).

### PK analysis

**Fig 4:**
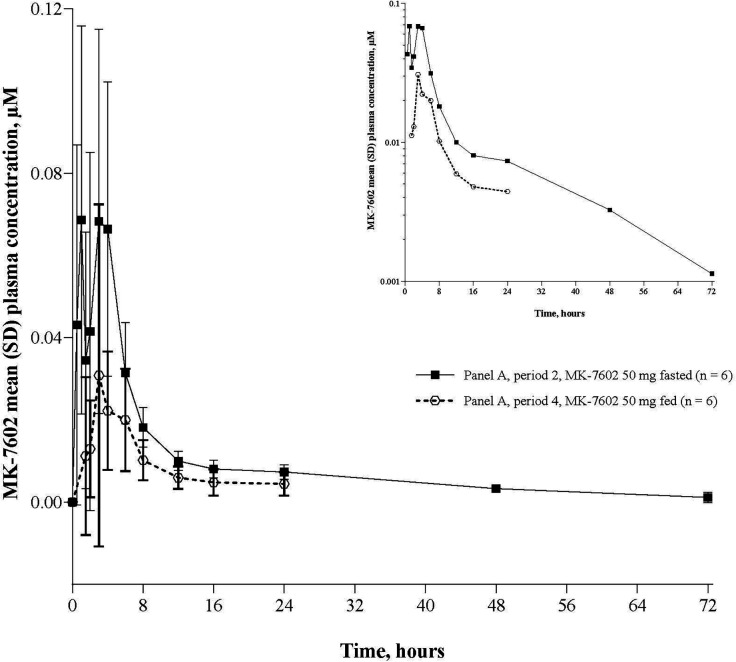
Arithmetic mean (± SD) concentration–time profiles of MK-7602 following administration of a single oral dose of MK-7602 to healthy adult male participants under fasted and fed states in panel A, linear scale. Insert: semi-log scale. SD, standard deviation.

**Fig 5:**
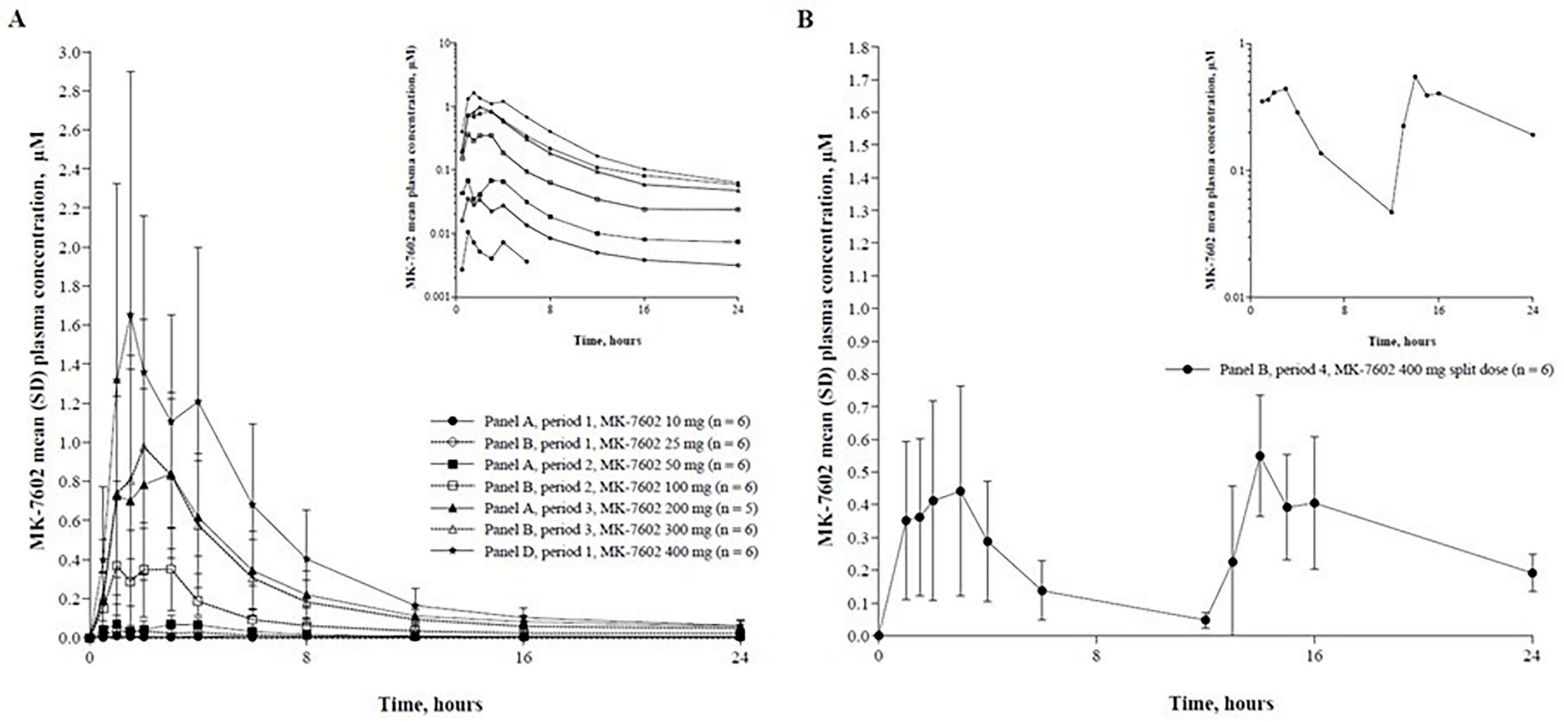
(**A**) Arithmetic mean (± SD) plasma concentration–time profiles of MK-7602 following administration of oral single-ascending doses of MK-7602 to healthy men in panel A, panel B, and panel D of study 7602-001, part 1, linear scale. Insert: semi-log scale. (**B**) Arithmetic mean (± SD) plasma concentration–time profiles of MK-7602 after split oral doses of MK-7602 400 mg (200 mg Q12H) to healthy men in panel B of study 7602-001, part 1, linear scale. Insert: semi-log scale. Q12H, every 12 h; SD, standard deviation.

In 7602-001, MK-7602 had a median time to maximum concentration (*T*_max_) of 1.8–2.5 h in fasted conditions, 3.6 h (range: 1.03–6.00) after a high-fat meal, and 8.5 h (range: 1.03–16.00) for the 400 mg split dose. The terminal half-life ranged from 23.2 to 41.2 h for fasted doses (50–400 mg) (Table 4). Plasma exposure (AUC, *C*_max_) increased in a roughly dose-proportional manner from 50 to 400 mg, with slopes of 1.17 (95% CI: 0.84–1.49) for AUC_0-∞_ and 1.18 (95% CI: 0.85–1.51) for *C*_max_. After a high-fat meal, *C*_max_, *C*_24_, and AUC*_0-∞_* were lower (GMRs of 0.28, 0.69, and 0.41, respectively) for the 50 mg dose (Fig. 4). Plasma concentration –time profiles of MK-7602 following the administration of single-ascending doses in panels A, B, and D from part 1 are shown in Fig. 5A and Fig. S1, and plasma concentration–time profiles following the administration of split doses of MK-7602 400 mg (200 mg Q12H) in panel B from part 1 are shown in Fig. 5B and Fig. S2.

**Fig 6:**
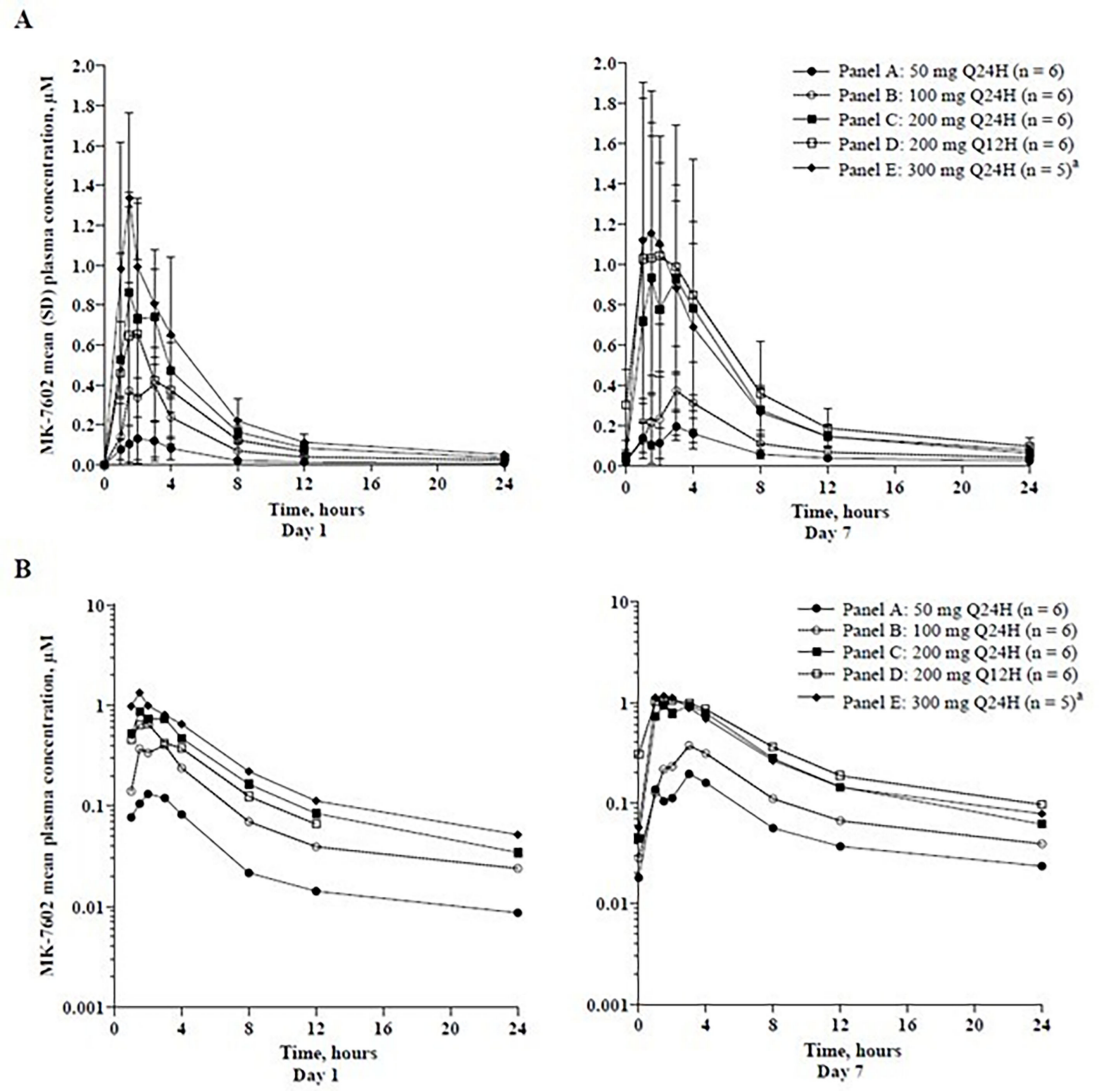
Arithmetic mean (± SD) plasma concentration–time profiles of MK-7602 following administration of multiple oral doses of MK-7602 to healthy participants in panels A, B, C, D, and E in study 7602-002. (**A**) Linear scale, (**B**) semi-log scale. ^a^Day 7 concentration data were not available for one participant in panel E. Q12H, every 12 h; Q24H, every 24 h; SD, standard deviation.

In 7602-002, MK-7602 had a median *T*_max_ of 1.5–3.0 h and terminal half-life of 31.3–41.4 h (Table 5). The accumulation ratio (day 7/day 1) for AUC_0-tau_ in Q24H dosing ranged from 1.03 to 2.20, with 3.09 for the 200 mg Q12H dose. *C*_trough_ ratios ranged from 0.83 to 2.02 (Table 5). Plasma concentration–time profiles of MK-7602 following administration of multiple doses in panels A, B, C, D, and E are shown in Fig. 6 and Fig. S3.

**TABLE 5:** Summary statistics of plasma MK-7602 PK after administration of multiple doses of MK-7602 in 7602-002^*a*^^,^^*e*^

PK parameter	MK-7602 50 mg Q24H		MK-7602 100 mg Q24H		MK-7602 200 mg Q24H		MK-7602 200 mg Q12H		MK-7602 300 mg Q24H	
	*n*	GM (95% CI)	*n*	GM (95% CI)	*n*	GM (95% CI)	*n*	GM (95% CI)	*n*	GM (95% CI)
Day 1 (first dose)										
AUC_0-tau_, µM·h^*b*^	6	0.643 (0.428–0.965)	6	1.75 (1.17–2.63)	6	4.57 (3.05–6.87)	6	1.81 (0.678–4.84)	6	6.18 (4.11–9.28)
*C*_max_, µM^*b*^	6	0.140 (0.0864–0.226)	6	0.422 (0.261–0.681)	6	1.03 (0.639–1.67)	6	0.497 (0.195–1.27)	6	1.36 (0.843–2.20)
*C*_trough_, µM^*b*^	6	0.00811 (0.00595–0.0111)	6	0.0208 (0.0152– 0.0283)	6	0.0342 (0.0251– 0.0465)	6	0.201 (0.125– 0.324)	6	0.0498 (0.0365– 0.0678)
*T*_max_, hours^*c*^	6	3.00 (1.02, 4.02)	6	3.00 (1.80, 3.00)	6	2.08 (1.08, 3.00)	6	2.08 (1.02, 3.00)	6	1.50 (1.02, 4.02)
Day 7 (last dose)										
	*n*	GM (95% CI)	*n*	GM (95% CI)	*n*	GM (95% CI)	*n*	GM (95% CI)	*n*	GM (95% CI)
AUC_0-tau_, µM·h^*b*^	6	1.41 (0.940–2.12)	6	2.32 (1.54–3.48)	6	6.42 (4.27–9.64)	6	5.59 (2.09–14.9)	5	6.36 (4.12–9.81)
*C*_max_, µM^*b*^	6	0.248 (0.153–0.401)	6	0.383 (0.237–0.618)	6	1.22 (0.755–1.97)	6	1.00 (0.393–2.57)	5	1.13 (0.676–1.89)
*C*_trough,_ µM^*b*^	6	0.0230 (0.0169– 0.0313)	6	0.0370 (0.0272– 0.0504)	6	0.0594 (0.0436– 0.0809)	6	0.167 (0.103– 0.268)	5	0.0716 (0.0514– 0.0996)
*T*_max_, hours^*c*^	6	3.00 (1.02, 4.05)	6	3.00 (1.03, 4.02)	6	2.08 (1.50, 4.02)	6	1.26 (1.02, 2.08)	5	1.50 (1.02, 2.08)
	*n*	GM (%GCV)	*n*	GM (%GCV)	*n*	GM (%GCV)	*n*	GM (%GCV)	*n*	GM (%GCV)
*t*_½_, hours	6	41.33 (27.73)	6	41.41 (25.44)	6	32.85 (18.72)	6	31.29 (20.09)	5	33.41 (25.87)
Accumulation ratio day 7/day 1										
		GMR (90% CI)		GMR (90% CI)		GMR (90% CI)		GMR (90% CI)		GMR (90% CI)
AUC_0-tau_, µM·h^*d*^		2.20 (1.59–3.04)		1.32 (0.96–1.83)		1.40 (1.01–1.94)		3.09 (1.17–8.17)		1.03 (0.72–1.46)
*C*_max_, µM^*d*^		1.78 (1.20–2.63)		0.91 (0.61–1.34)		1.18 (0.80–1.75)		2.02 (0.73–5.59)		0.83 (0.54–1.26)
*C*_trough_, µM^*d*^		2.83 (2.21–3.63)		1.78 (1.39–2.28)		1.74 (1.36–2.23)		0.83 (0.64–1.08)		1.44 (1.10–1.88)

^*a*^ AUC_0-12_, area under the curve from time 0 to 12 h; AUC_0-24_, area under the curve from time 0 to 24 h; AUC_0-tau_, area under the curve over the dosing interval; CI, confidence interval; *C*_max_, maximum concentration; *C*_trough_, trough concentration at either 12 or 24 h; GCV, geometric coefficient of variation; GM, geometric mean; GMR, geometric mean ratio; LS, least squares; Q12H, every 12 h; Q24H, every 24 h; *t*½, half-life; *T*_max_, time to maximum concentration.

^*b*^ Back-transformed LS mean and 95% CI from linear mixed-effects model performed on natural log-transformed values.

^*c*^ Median (minimum, maximum) reported for *T*_max_ (hours).

^*d*^ Back-transformed LS mean difference and 90% CI from linear mixed-effects model performed on natural log-transformed values.

^*e*^ The square root of the conditional mean squared error (residual error) from the linear mixed-effects model was as follows: 0.835 for AUC_0-12_ (μM·h), 0.326 for AUC_0-24_ (μM·h), 0.874 for *C*_max12_ (μM), 0.393 for *C*_max24_ (μM), 0.227 for *C*_trough12_ (μM), and 0.248 for *C*_trough24_ (μM). When multiplied by 100, these values yielded the pooled within-subject coefficient of variation estimates. One participant on the 300 mg dosing regimen had an incomplete concentration profile. Therefore, the day 7 concentration data for this participant were excluded from the analysis.

Plasma exposures on day 7 were comparable to those on day 3, indicating that steady-state exposures were reached by day 3. AUC_0-tau_ and *C*_max_ increased dose proportionally for 50 to 300 mg Q24H doses, whereas *C*_trough_ increases were less than dose proportional (slopes: AUC_0-tau_ = 0.97 [95% CI: 0.68–⁠1.26]; *C*_max_ = 1.01 [95% CI: 0.67–⁠1.35]; *C*_trough_ = 0.66 [95% CI: 0.47–⁠0.86]).

### Drug-drug interaction

**Fig 7:**
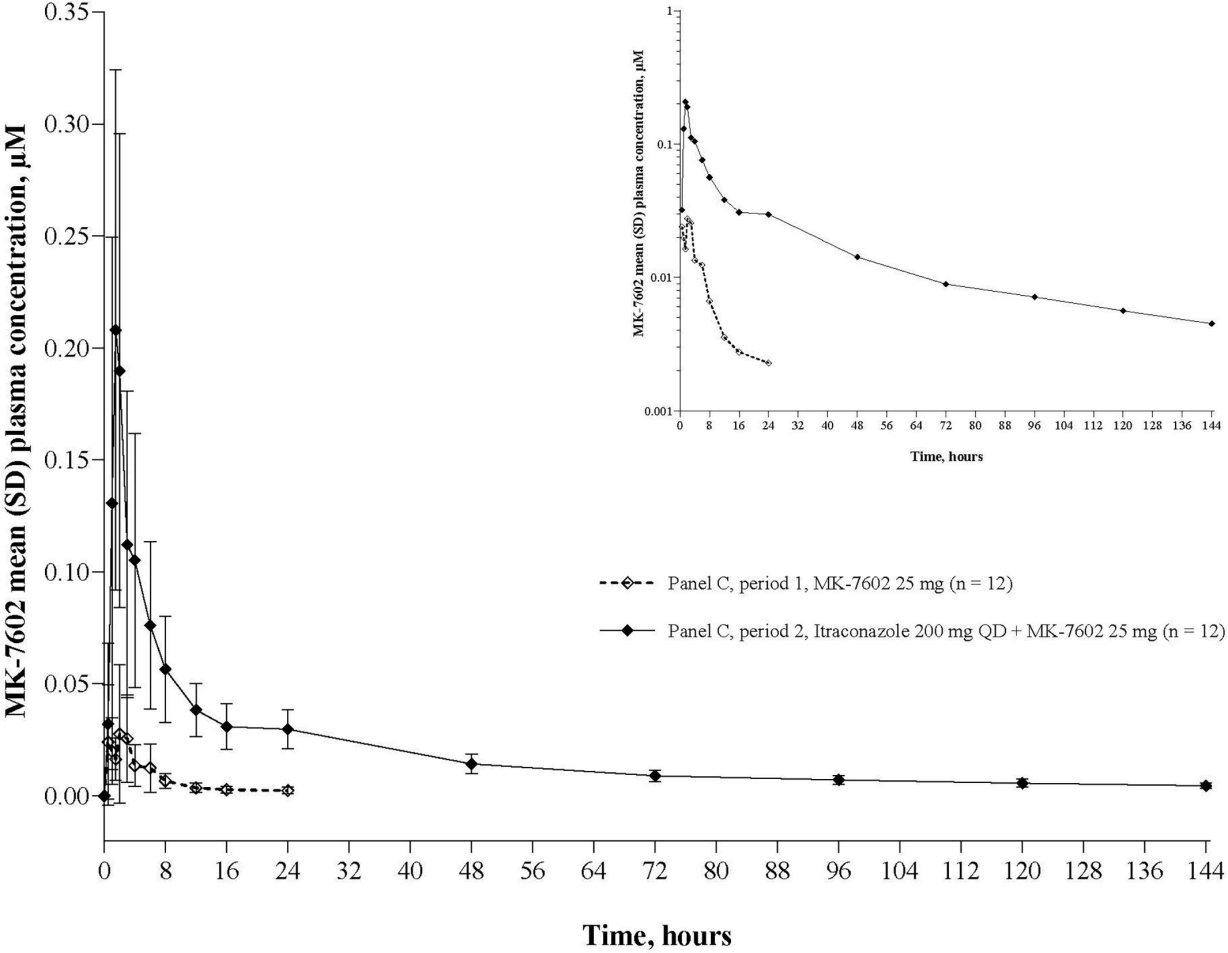
Arithmetic mean (± SD) concentration–time profiles of MK-7602 following administration of a single oral dose of MK-7602 alone and coadministered with itraconazole to healthy adult male participants in panel C, linear scale. Insert: semi-log scale. SD, standard deviation.

Coadministration of MK-7602 25 mg with itraconazole 200 mg in 7602-001 was associated with higher *C*_max_ and AUC*_0_*_-∞_ values (GMRs: 6.40 and 12.5, respectively), and a longer half-life (75.1 h; percentage geometric coefficient of variation [% GCV]: 30.3 vs 17.7 h [% GCV: 144.7]) compared with MK-7602 25 mg alone (Table 6, Fig. 7).

**TABLE 6:** Comparison of plasma PK of MK-7602 after single-dose oral administration of MK-7602 25 mg and MK-7602 25 mg + itraconazole 200 mg QD in 7602-001^*a*^

PK parameter	MK-7602 25 mg alone		MK-7602 25 mg + itraconazole 200 mg QD		MK-7602 25 mg + itraconazole 200 mg QD/ MK-7602 alone	%CV^*b*^
	*n*	GM (95% CI)	*n*	GM (95% CI)	GMR (90% CI)	
AUC_0-tau_, µM·h^*c*^	12	0.147 ~(0.0994–0.219)	12	1.27 (0.967–1.66)	8.59 (6.91–10.7)	29.7
AUC_0-∞_^*c*^	12	0.237 (0.145–0.386)	12	2.95 (2.38–3.64)	12.5 (9.23–16.8)	40.8
*C*_max_^*c*^	12	0.0347 (0.0202–0.0597)	12	0.222 (0.163–0.302)	6.40 (4.45–9.21)	49.7
*C*_24_, µM^*c*^	12	0.00286 (0.00230–0.00355)	12	0.0286 (0.0235–0.0347)	9.99 (8.24–12.1)	22.1
*T*_max_, hours^*d*^	12	2.08 (0.50, 6.00)	12	1.50 (1.00–2.13)		
	*n*	GM (%GCV)	*n*	GM (%GCV)		
Apparent *t*_½_, hours	12	17.7 (144.7)	12	75.1 (30.3)		

^*a*^ %CV, percentage coefficient of variation; AUC_0-∞_, area under the concentration-time curve to infinity; AUC_0-tau_, area under the curve to time tau; *C*_24_, concentration at 24 h; CI, confidence interval; *C*_max_, maximum concentration; GM, geometric mean; LS, least squares; PK, pharmacokinetics; sqrt, square root; QD, twice daily; *t*½, half-life; *T*_max_, time to maximum concentration.

^*b*^ The pseudo within-subject %CV was calculated using the following formula: 100 × sqrt[(σ²A + σ²B - 2σAB)/2], where *σ²A *and *σ²B *are the estimated variances on the log scale for the two treatment groups, and σAB is the estimated covariance, each obtained from the linear mixed-effects model.

^*c*^ Back-transformed LS mean and 95% CI from linear mixed-effects model performed on natural log-transformed values.

^*d*^ Median (minimum, maximum) reported for *T*_max_ (hours).

### Renal excretion (study 7602-001)

The GM fraction of MK-7602 excreted in urine ranged from 1.83% (SD: 13.0) for the 50 mg dose to 2.81% (SD: 102.8) for the 300 mg dose. The amount of MK-7602 excreted unchanged in urine from time zero to 24 h post dose was 0.916 mg (SD: 13.0) for the 50 mg dose and 8.43 mg (SD: 102.8) for the 300 mg dose. Renal clearance values were 3.66 L/h (SD: 13.1) for the 50 mg dose and 3.84 L/h (SD: 16.2) for the 300 mg dose.

## DISCUSSION

Antimalarial resistance remains a global health challenge, and there is an urgent need for antimalarial drugs with novel mechanisms of action. MK-7602 is a first-in-class dual-plasmepsin IX/X inhibitor being developed for the treatment of uncomplicated *P. falciparum* malaria. A clinical candidate for malaria treatment should be well tolerated and effective within a short period of dosing (3 days or fewer). This article is the first description of the safety, tolerability, and PK of single and multiple doses of MK-7602 in healthy adult participants.

The primary PK hypothesis, informed by efficacious concentrations of MK-7602 in a preclinical mouse model of malaria (10), was met in both studies, demonstrating that MK-7602 consistently reached target plasma concentrations across various dosing regimens. Under fasted conditions, MK-7602 showed rapid absorption. Absorption was delayed and resulted in lower plasma exposure following a high-fat meal. AUC_0-tau_ and *C*ₘₐₓ increased dose proportionally within the 50–400 mg dose range, whereas *C*_trough_ showed less than proportional increases, suggesting that further investigation into factors influencing *C*_trough_ dynamics is warranted. Steady-state exposures were reached by day 3, as indicated by the observed accumulation ratios, reflecting stable PK properties throughout the dosing period.

The data suggest that renal clearance is not a major elimination pathway for MK-7602, as indicated by the low fraction excreted in urine. A definitive assessment of elimination pathways in humans will be conducted in future studies.

Coadministration with itraconazole, a strong cytochrome P450 3A (CYP3A) and P-glycoprotein (P-gp) inhibitor, resulted in increases in *C*_max_ and AUC*_0_*_-∞_ by approximately 6-fold and 12-fold, respectively, consistent with the involvement of CYP3A in the metabolism of MK-7602 and transport via P-gp. The impact of moderate and/or strong CYP3A inducers on the PK of MK-7602 will be evaluated in future studies.

Single and multiple doses of MK-7602 up to 400 mg, including coadministration of MK-7602 with itraconazole 200 mg, were generally well tolerated in healthy participants in these two phase 1 studies. No clinically meaningful trends were observed in laboratory tests, vital signs, or ECGs, and no serious AEs or deaths were reported in these studies. Several cases of diarrhea were observed during 7602-001 part 2, period 2, with coadministration of MK-7602 and itraconazole, with 50% of participants experiencing this AE, and no cases of diarrhea following administration of MK-7602 alone. Given that itraconazole is known to be associated with gastrointestinal side effects, including diarrhea (12), this finding can likely be attributed to the combined treatment regimen rather than to MK-7602 alone.

These studies provide detailed characterization of the safety and clinical PK of MK-7602 across a range of single and multiple doses. Some potential limitations should be noted. The relatively small sample size of both studies should be considered when interpreting safety data, as this may limit the detection of less common AEs. Additionally, the limited diversity in ethnicities and demographics, including sex (only male participants were enrolled in 7602-001 and 77% of the participants in 7602-002 were male), as well as the restricted weight range of the study population in 7602-001, may impact the generalizability of the findings. More safety data will be collected in larger studies enrolling the relevant patient populations.

The assessment of the effect of a high-fat meal in 7602-001 part 1 used 50 mg as the reference dose, and the reference dose studied in the itraconazole drug-drug interaction assessment in 7602-001 part 2 was 25 mg. Doses of MK-7602 below 50 mg are outside of the linear range of exposures, which could limit the generalizability of the results observed in these studies to higher dose levels. Additionally, exposures of antimalarial drugs in healthy participants can differ significantly from exposures in individuals infected with malaria (13, 14). In future clinical studies, it will be important to further characterize MK-7602 exposures under relevant physiological conditions, including infection.

Overall, MK-7602 was well tolerated following single and multiple doses. PK data indicated rapid absorption under fasted conditions, dose-proportional increases in *C*ₘₐₓ and AUC_0-tau_ between 50 and 400 mg, and increased concentrations when coadministered with a CYP3A and P-gp inhibitor. These findings support further clinical development of MK-7602, which is currently being evaluated in a human malaria challenge study (NCT06294912) to inform dose selection for future studies in patients with *P. falciparum* malaria.

## Data Availability

The data sharing policy, including restrictions, of Merck Sharp & Dohme LLC, a subsidiary of Merck & Co., Inc., Rahway, NJ, USA, is available at https://trialstransparency.msdclinicaltrials.com/policies-perspectives.aspx. Requests for access to the clinical study data can be submitted via email to the Data Access mailbox (mailto:dataaccess@msd.com).

## SUPPLEMENTAL MATERIAL

Supplemental material aac.01261-25-s0001.docx Fig. S1 to S3; Tables S1 to S6.

ASM does not own the copyrights to Supplemental Material that may be linked to, or accessed through, an article. The authors have granted ASM a non-exclusive, world-wide license to publish the Supplemental Material files. Please contact the corresponding author directly for reuse.
