# Supplementary material for: First-in-human safety and pharmacokinetics of MK-7602, the antimalarial inhibitor of plasmepsins IX/X, in single- and multiple-ascending-dose studies
Source: Antimicrob Agents Chemother. 2026 Jan 14;70(2):e01261-25. doi: 10.1128/aac.01261-25 (PMC12888855; doi:10.1128/aac.01261-25)
Supplement: Supplemental material — Fig. S1 to S3; Tables S1 to S6. [file aac.01261-25-s0001.docx]

# **SUPPLEMENTARY MATERIAL**

## **Supplementary Appendix A.** High-fat breakfast composition for panel A, period 4 in study 7602-001 part 1

*Study 7602-001: High-fat breakfast for fed panel A, period 4*

- Prior to administration of study drug, a standard high-fat breakfast was provided, which included
  - 2 fried or scrambled eggs, 2 strips of bacon (or 60 g of feta cheese for vegetarians)
  - 2 slices of toast with butter, 4 oz of hash browns
  - 240 mL whole milk
- Breakfast was consumed entirely within 30 minutes; start and stop times were recorded

**Fig. S1.** Arithmetic mean (± SD) plasma concentration versus time profiles of MK-7602 following administration of oral single-ascending doses of MK-7602 to healthy adult men in panels A, B, and D of part 1; linear scale. Insert: semi-log scale. SD, standard deviation.


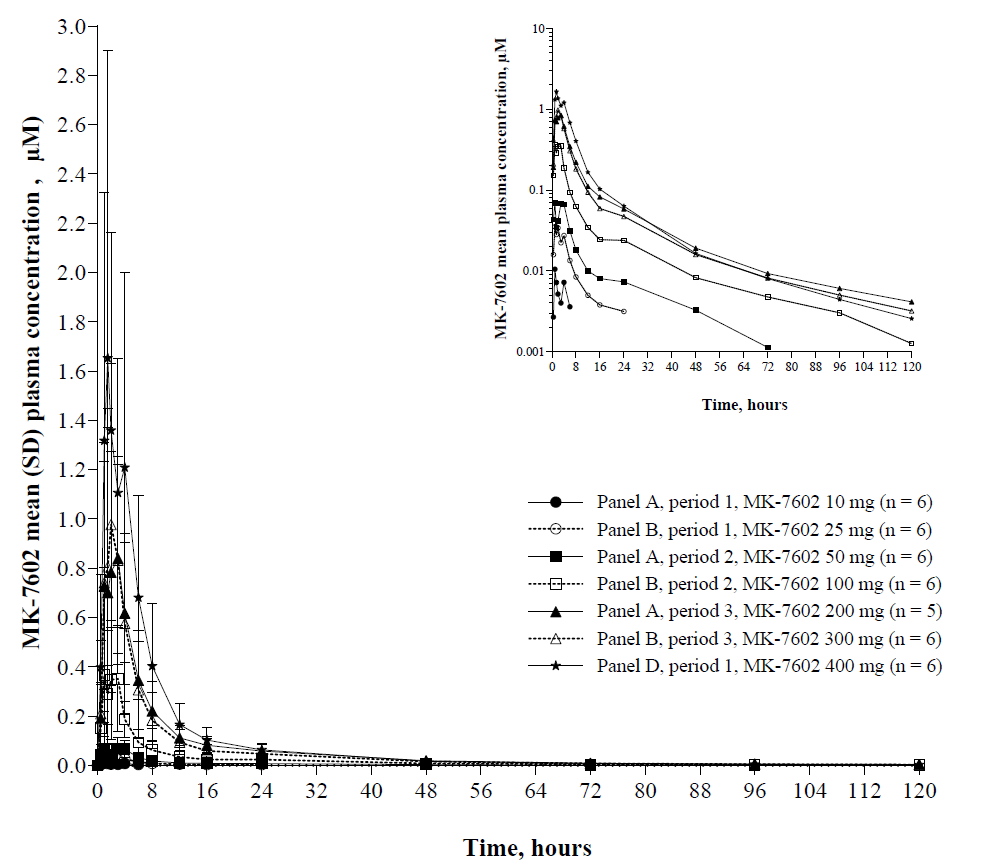


**Fig. S2.** Arithmetic mean (± SD) plasma concentration versus time profiles of MK-7602 following administration of split oral doses of MK-7602 400 mg (200 mg Q12H) to healthy men in panel B, part 1; linear scale. Insert: semi-log scale. Q12H, every 12 hours; SD, standard deviation.


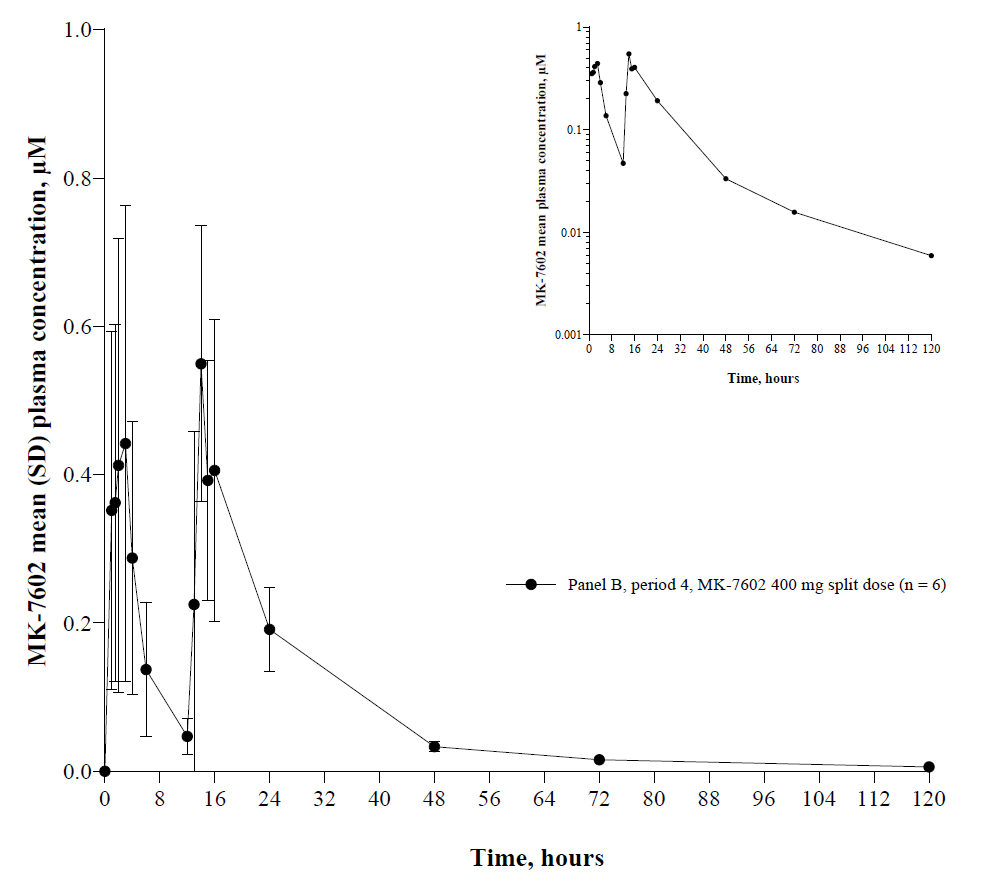


**Fig. S3.** Arithmetic mean (± SD) plasma concentration versus time profiles of MK-7602 following administration of multiple oral doses of MK-7602 to healthy participants in panels A, B, C, D, and E in study 7602-002. (A) Linear scale, (B) semi-log scale. ^a^Day 7 concentration data were not available for one participant in panel E. Q12H, every 12 hours; Q24H, every 24 hours; SD, standard deviation.

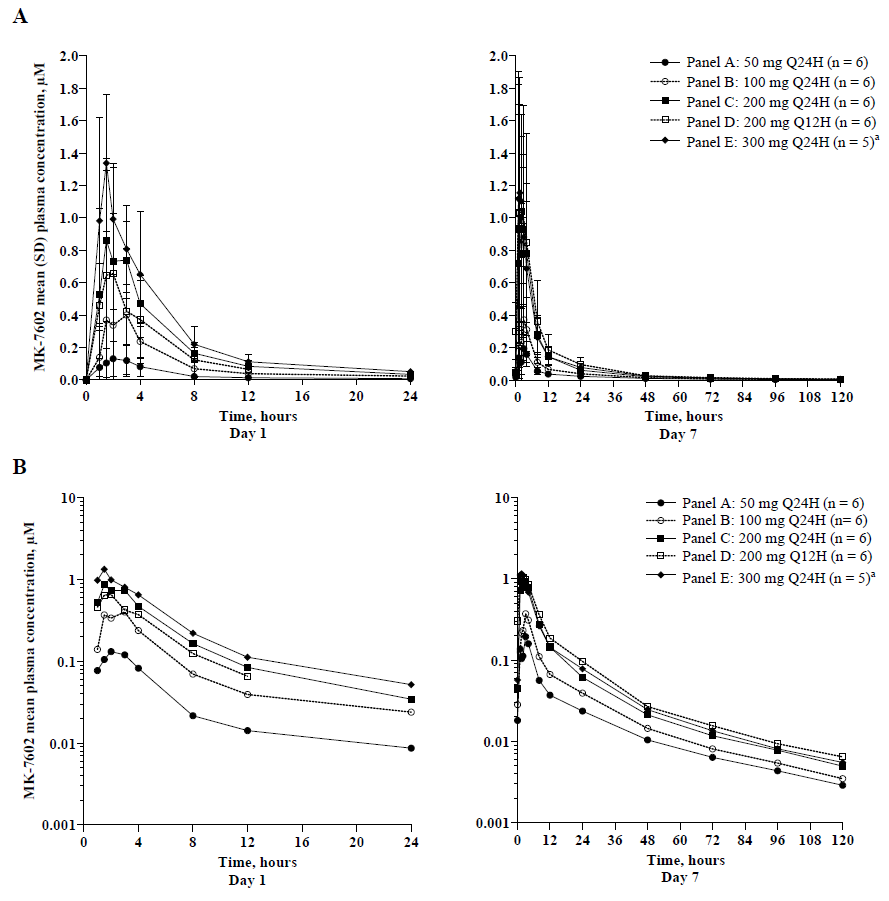


**TABLE S1.** Exclusion criteria for studies 7602-001 and 7602-002^a^

| Exclusion criteria for studies 7602-001 and 7602-002 |
| --- |
| History of clinically significant medical conditions affecting the endocrine, gastrointestinal, cardiovascular, hematological, hepatic, immune, renal, respiratory, genitourinary, or major neurological systems, including stroke or chronic seizures |
| Mental or legal incapacitation, significant emotional issues at screening or expected during the study, or history of clinically significant psychiatric disorder in the past 5 years. Participants with situational depression can be enrolled at the investigator’s discretion |
| History of cancer unless adequately treated, deemed cured, and unlikely to recur per the study investigator’s opinion |
| An estimated glomerular filtration rate of ≤80 mL/min/1.73 m^2^ |
| History of severe allergies or anaphylactic reaction |
| Positive test results for hepatitis B, hepatitis C, or HIV |
| Any major surgery or loss of 500 mL of blood within the past 4 weeks |
| Use of medications (prescription, nonprescription, or herbal remedies) within 2 weeks (or 5 half-lives) before or use of CYP3A and P-gp inducers (e.g., rifampin, St. John’s Wort) within 4 weeks before the study, and throughout the study, including washout periods, and up to 2 weeks after the last dose |
| Hypersensitivity to itraconazole or other azole antifungal agents*^b^* |
| Has participated in another investigational study within 4 weeks (or 5 half-lives, whichever is longer) before the screening visit, with the window based on the date of the last visit in the previous study |
| Meets any of the following cardiac parameters: QTc interval ≥450 ms (males) or ≥460 ms (females), history of risk factors for torsades de pointes (e.g., heart failure/cardiomyopathy or family history of long QT syndrome), uncorrected hypokalemia or hypomagnesemia, or is taking concomitant medications that prolong the QT/QTc interval |
| Under the age of legal consent |
| Smoker or has used nicotine or nicotine-containing products (e.g., nicotine patch and electronic cigarette) within 3 months before screening |
| Consumes more than three servings of alcoholic beverages (one serving is approximately equivalent to beer [354 mL/12 oz], wine [118 mL/4 oz], or distilled spirits [29.5 mL/1 oz]) per day. Participants who consume four servings of alcoholic beverages per day can be enrolled at the discretion of the investigator |
| Consumes excessive amounts, defined as more than six servings (one serving is approximately equivalent to 120 mg caffeine) of coffee, tea, cola, energy drinks, or other caffeinated beverages per day |
| Regular cannabis or illicit drug user, or history of drug (including alcohol) abuse within approximately 12 months. Participants must have a negative drug screen before randomization |
| The investigator has concerns about safe participation or deems the participant inappropriate for the study |
| The participant or their immediate family member (e.g., spouse, parent, sibling, child) are investigational site or sponsor staff involved in the study |

*^a^*CYP3A, cytochrome P450 3A; *P-gp,* P-glycoprotein; QTc, corrected QT interval.

*^b^*Applicable to part 2 of 7602-001.

## **TABLE S2.** Summary of AEs in 7602-001 part 1^a^

|  | **x** | **MK-7602 25 mg** | **MK-7602 50 mg** | **MK-7602 100 mg** | **MK-7602 200 mg** | **MK-7602 300 mg** |  |
| --- | --- | --- | --- | --- | --- | --- | --- |
| Participants in population, n | 6 | 6 | 6 | 6 | 5 | 6 |  |
| With one or more AEs | 6 (100) | 6 (100) | 4 (66.7) | 5 (83.3) | 3 (60.0) | 3 (50.0) |  |
| With no AE | 0 | 0 | 2 (33.3) | 1 (16.7) | 2 (40.0) | 3 (50.0) |  |
| With drug-related*^b^* AEs | 4 (66.7) | 3 (50.0) | 1 (16.7) | 1 (16.7) | 0 | 0 |  |
| With nonserious AEs | 6 (100) | 6 (100) | 4 (66.7) | 5 (83.3) | 3 (60.0) | 3 (50.0) |  |
|  | **MK-7602 50 mg (fed)** | **MK-7602  400 mg split dose (Q12H)** | **MK-7602 400 mg** | **MK-7602 total*^c^*** | **Placebo*^d^*** | **Total** | **Total follow-up** |
| Participants in population, n | 6 | 6 | 6 | 22 | 16 | 24 | 24 |
| With one or more AEs | 5 (83.3) | 4 (66.7) | 5 (83.3) | 20 (90.9) | 11 (68.8) | 22 (91.7) | 1 (4.2) |
| With no AE | 1 (16.7) | 2 (33.3) | 1 (16.7) | 2 (9.1) | 5 (31.3) | 2 (8.3) | 23 (95.8) |
| With drug-related*^b^* AEs | 2 (33.3) | 1 (16.7) | 2 (33.3) | 10 (45.5) | 3 (18.8) | 11 (45.8) | 0 |
| With nonserious AEs | 5 (83.3) | 4 (66.7) | 5 (83.3) | 20 (90.9) | 11 (68.8) | 22 (91.7) | 1 (4.2) |

*^a^*AE, adverse event; Q12H, every 12 hours.

*^b^*Determined by the investigator to be related to the drug.

*^c^*MK-7602 total column is pooled across panels A, B, and D.

*^d^*Placebo column is pooled across panels A, B, and D.

Values are expressed as n (%) unless otherwise noted.

Panel A: placebo and MK-7602 10 mg, 50 mg, 200 mg, and 50 mg (with food); panel B: placebo and MK-7602 25 mg, 100 mg, 300 mg, and a 400-mg split dose (200 mg Q12H); panel D: placebo and MK-7602 400 mg. No participants experienced serious AEs, had serious drug-related AEs, died during the study, died due to a drug-related AE, discontinued the study due to an AE, discontinued the drug due to a drug-related AE, discontinued the drug due to a serious AE, or discontinued the drug due to a serious drug-related AE.

## **TABLE S3.** Summary of AEs in 7602-001 part 2*^a^*

|  | **MK-7602 25 mg alone** | **Itraconazole 200 mg QD + MK-7602 25 mg** | **Total** | **Total follow-up** |
| --- | --- | --- | --- | --- |
| Participants in population, n | 12 | 12 | 12 | 12 |
| With one or more AEs | 7 (58.3) | 10 (83.3) | 1 (8.3) | 11 (91.7) |
| With no AEs | 5 (41.7) | 2 (16.7) | 11 (91.7) | 1 (8.3) |
| With drug-related*^b^* AEs | 5 (41.7) | 6 (50.0) | 0 | 8 (66.7) |
| With nonserious AEs | 7 (58.3) | 10 (83.3) | 1 (8.3) | 11 (91.7) |

^a^AE, adverse event; QD, once daily.

*^b^*Determined by the investigator to be related to the drug.

Values are expressed as n (%) unless otherwise noted.

Panel C: MK-7602 25 mg and itraconazole 200 mg. No participants experienced serious AEs, had serious drug-related AEs, died during the study, died due to a drug-related AE, discontinued the study due to an AE, discontinued the drug due to a drug-related AE, discontinued the drug due to a serious AE, or discontinued the drug due to a serious drug-related AE.

##

## **TABLE S4.** Model-based GM and 95% CI for MK-7602 C_24_ by dose with posterior probability of plasma fasted GM C_24_ ≥0.017 μM (single ascending dose; 7602-001)*^a^*

| **Dose, mg** | **n** | **C_24_, µM** | **95% CI** | **Posterior probability, %** | **Participants satisfying C_24_ target, %** |
| --- | --- | --- | --- | --- | --- |
| 10 | 6 | 0.0034 | 0.0012–0.0095 | 2.29 | 0.00 |
| 25 | 6 | 0.0038 | 0.0025–0.0058 | 0.76 | 0.00 |
| 50 | 6 | 0.0073 | 0.005–0.0106 | 3.07 | 0.00 |
| 100 | 5 | 0.0223 | 0.0147–0.034 | 78.47 | 80.00 |
| 200 | 5 | 0.0533 | 0.0351–0.0809 | 98.07 | 100 |
| 300 | 6 | 0.0443 | 0.0305–0.0644 | 97.44 | 100 |
| 400 | 6 | 0.0551 | 0.0384–0.0789 | 98.76 | 83.33 |

*^a^*C_24_, concentration at 24 hours; CI, confidence interval; COVID-19, coronavirus disease 2019; GM, geometric mean; LOQ, limit of quantification.

C_24_ was below the LOQ for one participant receiving 100 mg. One participant in panel A missed a dose (200 mg, period 3) due to a positive COVID-19 test.

## **TABLE S5**. Model-based GM and 95% CI for MK-7602 C_24_ by dose (Q24H) and day with posterior probability of plasma fasted GM C_24_ ≥0.017 μM in 7602-002*^a^*

| **MK-7602 dose, mg** | **Day** | **n** | **C_24_, µM** | **95% CI** | **Posterior probability, %** | **Participants satisfying C_24_ target, %** |
| --- | --- | --- | --- | --- | --- | --- |
| 50 | 1 | 6 | 0.0081 | 0.00627–0.0105 | 0.14 | 0.00 |
| 50 | 7 | 6 | 0.0230 | 0.0178–0.0297 | 95.34 | 100 |
| 100 | 1 | 6 | 0.0208 | 0.0160–0.0268 | 88.42 | 66.67 |
| 100 | 7 | 6 | 0.0370 | 0.0286–0.0479 | 99.89 | 100 |
| 200 | 1 | 6 | 0.0342 | 0.0264–0.0442 | 99.82 | 100 |
| 200 | 7 | 6 | 0.0594 | 0.0459–0.0769 | 99.99 | 100 |
| 300 | 1 | 6 | 0.0498 | 0.0385–0.0644 | 99.98 | 100 |
| 300 | 7 | 5 | 0.0716 | 0.0544–0.0942 | 99.98 | 100 |

*^a^*C_24_, concentration at 24 hours; CI, confidence interval; GM, geometric mean; Q24H, every 24 hours.

One participant in the 300-mg dosing regimen had an incomplete concentration profile; therefore, day 7 concentration data for this participant were not included in the analysis.

## **TABLE S6**. Model-based GM and 95% CI for MK-7602 C_12_ by dose (Q12H) and day with posterior probability of plasma fasted GM C_12_ ≥0.017 μM (7602-002)*^a^*

| **MK-7602 dose, mg** | **Day** | **n** | **C_12_, µM** | **95% CI** | **Posterior probability, %** | **Participants satisfying C_12_ target, %** |
| --- | --- | --- | --- | --- | --- | --- |
| 200 | 1 | 6 | 0.2010 | 0.137–0.294 | 100 | 100 |
| 200 | 7 | 6 | 0.1670 | 0.114–0.243 | 100 | 100 |

*^a^*C_12_, concentration at 12 hours; CI, confidence interval; GM, geometric mean; Q12H, every 12 hours.
